# Forensic age estimation at the 15- and 18-year thresholds using MRI of the wrist: application of the Vieth staging method

**DOI:** 10.1007/s00414-026-03785-2

**Published:** 2026-04-08

**Authors:** Ozkan Alatas, Rıdvan Binici, Onur Sari, Murat Serdar Gurses, Busra Has, Umit Belet

**Affiliations:** 1 Department of Radiology, İzmir Faculty of Medicine, University of Health Sciences, İzmir, Turkey; 2 Private Researcher, Sakarya, Turkey; 3 Department of Radiology, Faculty of Medicine, Düzce University, Düzce, Turkey

**Keywords:** Distal radial epiphsis, Forensic age estimation, Magnetic resonance imaging, Vieth staging method

## Abstract

**Objectives:**

The objective of this study was to assess distal radial epiphyseal fusion on MRI based on the Vieth staging method and to evaluate the relevance of the obtained findings for determining legal majority in forensic age estimation.

**Methods:**

Wrist images obtained via a 1.5 T MRI scanner were retrospctively evaluated by two independent observers in a study population of 516 cases (252 females, 264 males) aged 10–28 years, following the application of exclusion criteria. MR images were independently evaluated by two experienced radiologists, with intraobserver agreement assessed through repeat evaluation after one month and consensus assessment performed jointly by both observers. Intra- and interobserver agreement rates were evaluated using Cohen’s kappa and linear weighted kappa statistics. Descriptive statistics, including minimum, maximum, mean ± standard deviation, 95% confidence interval, and median, were defined for age distribution. Sex-specific age differences within each stage were analyzed using either the Student’s t-test or the Mann–Whitney U test.

**Results:**

Descriptive age statistics were evaluated separately for females and males. No significant sex-related age difference was observed at stage 2, whereas males were significantly older than females at all subsequent stages. Inter- and intraobserver agreement assessment was very good, with high agreement levels observed for both analyses.

**Conlusion:**

The findings indicate that the Vieth staging method applied to wrist MRI may be applicable for forensic age estimation at the 15- and 18-year legal thresholds in both sexes. However, further validation in diverse populations using balanced age groups and experienced observers is required.

## Introduction

Forensic age assessment is requested in many countries for various criminal and civil law purposes. Reasons for legal requirements include cases of insufficient or questionable birth records, child abuse, determining the age of legal responsibility, and illegal immigration [1, 2]. Economic crises, wars and forced displacement drive the increase in asylum applications to European Union countries. Age estimation is performed when individuals do not have valid identity documents in the countries they migrate to and have unreliable or incomplete official records. Accurately determining the age of migrant children is crucial for establishing their legal status and ensuring their protection [3]. In most European countries, the minimum age of criminal responsibility varies considerably, ranging from 7 to 21 years, with critical thresholds most frequently set at 14, 16, 18, and 21 years [4, 5].

The Study Group on Forensic Age Diagnostics of the German Society of Legal Medicine (AGFAD) recommends a comprehensive approach that includes physical examination, X-ray examination of the left hand, dental examination, and orthopantomography. When skeletal maturation of the hand is complete, additional radiological evaluation of the medial clavicular epiphysis using conventional radiography and/or computed tomography is advised [6]. To date, AGFAD has not recommended radiation-free imaging modalities such as magnetic resonance imaging (MRI) or ultrasonography for forensic age estimation. In practice, forensic age assessment relies on reference studies involving imaging methods that use ionising radiation. As forensic age estimation is frequently applied in age groups such as children and adolescents, who are highly sensitive to the adverse effects of radiation, existing studies based on radiation-free imaging methods are important from an ethical perspective [7–9].

There are several studies in the literature evaluating the knee, ankle, clavicle, and iliac crest using MRI [10–18]. In addition, MRI of the distal radial epiphysis has been assessed using various staging methods [19–24]. Vieth et al. described a novel staging method for epiphyseal-diaphyseal fusion of the knee joint using 3.0 T MRI. This staging method is based on the T1-weighted turbo spin-echo (T1-TSE) sequence and additionally incorporates the T2-TSE signal pre-saturation with inversion recovery (SPIR) sequence. In the first step, T1-TSE sequences are primarily used to evaluate epiphyseal–diaphyseal fusion, overall bone anatomy, and atypical conditions. During the second step, T2-TSE SPIR sequences are employed to identify water and fat components within bone and cartilage fragments, thereby refining the staging method [25]. Several studies have applied the Vieth staging method to long bones using MRI, including the shoulder, knee, and ankle [26–32]. However, only a limited number of studies have evaluated the distal radial epiphysis using MR images according to the Vieth staging method [33–35]. The primary aim of our study is to evaluate the maturation of the distal radial epiphysis using the Vieth method [15] on 1.5 T MR images and to determine its applicability in critical age ranges for forensic age estimation. The secondary aims were to compare our findings with previous studies that evaluated wrist MRI using the Vieth method [33–35], to determine sex-specific age estimates for the distal radius based on the minimum age concept, and to establish a reference database for future studies.

## Materials and methods

### Study population

This study was conducted retrospectively and included cases whose wrist MR images were obtained using a 1.5 T MRI scanner at the Clinic of Radiology, İzmir City Hospital between November 2023 and November 2025. The study was performed after obtaining approval from the ethics committee (approval number: 2026-66).

Based on the information retrieved from the Hospital Information Management System, cases with a history of chronic systemic and neoplastic diseases and related treatments (e.g., steroid treatment, hormonotherapy, chemo-radiotherapy), a history of wrist trauma or infection, or a history of wrist operation were excluded. Furthermore, cases in which motion artifacts negatively affected the evaluation of the images were also excluded from the study. As a consequence, the study population consisted of a total of 516 subjects (252 females & 264 males) aged between 10* and *28 years. The mean age was 20.47 years for females (standard deviation [SD] = 4.45) and 21.62 years for males (SD = 4.23). Table 1 shows the distribution of cases by age and sex.

**Table 1:** Distribution of cases by age and sex

Age	Female	Male	Total
10	2	2	4
11	3	3	6
12	4	3	7
13	8	4	12
14	8	3	11
15	15	6	21
16	16	13	29
17	15	20	35
18	16	12	28
19	11	17	28
20	26	14	40
21	18	12	30
22	16	24	40
23	22	32	54
24	21	20	41
25	13	20	33
26	12	29	41
27	16	22	38
28	10	8	18
Total	252	264	516

### MRI examination

Wrist MR images acquired on a 1.5 T MRI scanner (GE Signa, USA, 2022) using a dedicated coil (16-channel Tx/Rx flexible extremity coil) were utilized in this study. The distal radius epiphysis was evaluated on coronal T1- and fat-saturated (FS) proton density (PD)-weighted images. T1-TSE sequences in the coronal plane were used with parameters: TR 450 ms, TE 12 ms, slice thickness 2 mm, interslice gap 2 mm, field of view (FOV) 140 × 100 mm, matrix 384 × 256, voxel size 0.4 × 0.5 × 2.0 mm, scan time 1.11 min. FS PD-weighted TSE (FS PD-TSE) coronal plane with the following parameters TR 2400 ms, TE 50 ms, slice thickness 2 mm, interslice gap 2 mm, FOV 100 × 80 mm, matrix 384 × 256, voxel size 0.4 × 0.4 × 2.0 mm, scan time 1.35 min.

### Image interpretation

The MR images were independently evaluated on a high-resolution monitor in the workstation by two radiologist observers with 17 and 30 years of professional experience, respectively, who were blinded to the subjects’ age and sex. One month after the initial assessment, all cases were re-evaluated by the radiologist with 30 years of experience to assess intraobserver agreement. Finally, all cases were reviewed together by both observers to reach a consensus assessment.

The staging method used in our study by Vieth et al. [15], which they defined in T1-TSE and T2-TSE SPIR sequences, is as follow:

*‘Stage 2: In the T1-w sequence a continuous band of intermediate signal intensity is visible*,* walled by serrated lines of low to no signal intensity towards the epiphysis and the diaphysis. In the T2-w sequence*,* the epiphysis is demarcated by a serrated line of low to no signal intensity. The metaphysis shows two serrated lines of high signal intensity. Both lines can be continuous or discontinuous.*

*Stage 3: In the T1-w sequence a discontinuous band of intermediate signal intensity is visible. The band is walled by serrated lines of low to no signal intensity towards the epiphysis and the diaphysis that sporadically convene and interrupt the band*,* forming a single serrated line with no signal intensity. In the T2-w sequence*,* the metaphysis shows two serrated lines of high signal intensity that sporadically convene*,* forming a single thin and serrated line of high signal intensity.*

*Stage 4: In the T1-w sequence a discontinuous thin and serrated line of intermediate signal intensity between the epiphysis and the diaphysis is visible. In the continuity of the line*,* thicker sections with no signal intensity can be seen. In the T2-w sequence*,* a thin single*,* discontinuous or dotted line of the hyperintense signal is visible in the same position as the described thin line of the corresponding T1-w sequence. In the continuity of the line*,* thicker hyperintense sections can be seen.*

*Stage 5: In the T1-w sequence a continuous thin line of intermediate signal intensity between the epiphysis and the diaphysis is visible. The T2-w sequence shows a single thin*,* discontinuous or dotted line of hyperintense signal in the same position as the described thin line of the corresponding T1-w sequence.*

*Stage 6: In the T1-w sequence a continuous thin line of intermediate signal intensity between the epiphysis and the diaphysis is visible. The T2-w sequence shows no hyperintense signal in the same position as the described thin line of the corresponding T1-w sequence.’*

**Fig. 1:**
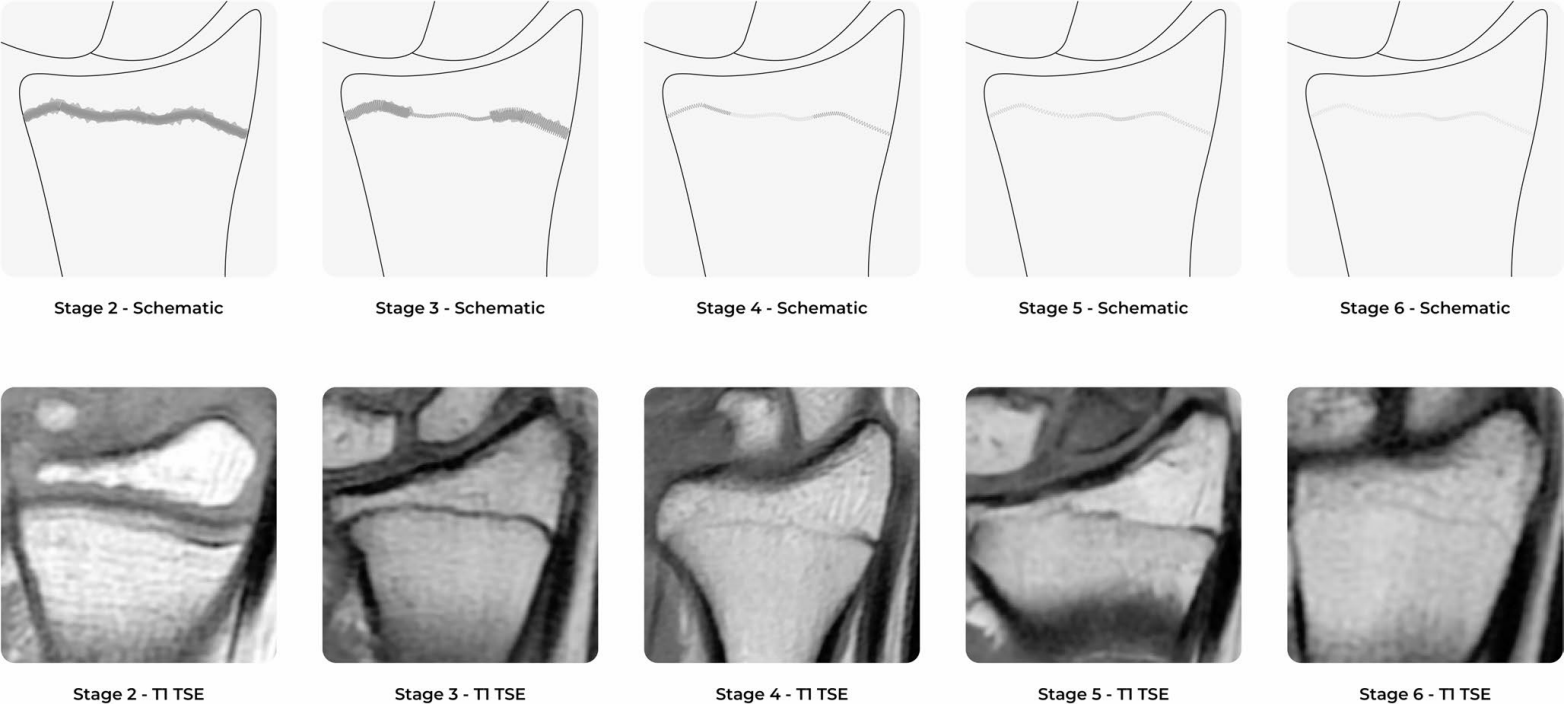
Schematic representations of the stages in the T1-TSE sequence together with representative MR images of the distal radial epiphysis in coronal orientation. From left to right: 14-year-old female, 15-year-old male, 17-year-old male, 19-year-old male, and 23-year-old male

**Fig. 2:**
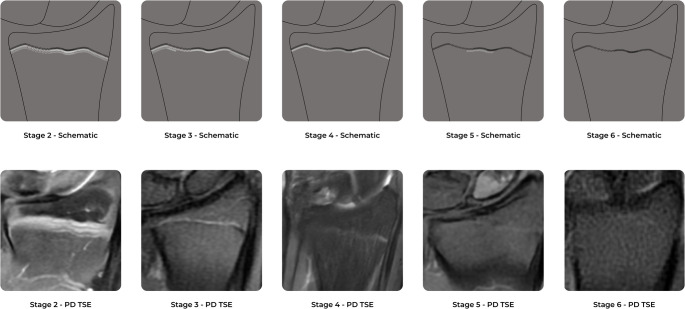
Schematic representations of the stages in the FS PD-TSE sequence with corresponding representative MR images of the distal radial epiphysis in coronal orientation. From left to right: 14-year-old female, 15-year-old male, 17-year-old male, 19-year-old male, and 23-year-old male

Schematic drawings of the stages in the T1-TSE and FS PD-TSE sequences, together with representative MR images of all stages of the distal radial epiphysis, are shown in Figs. 1 and 2.

### Statistical analysis

The age distribution of females and males were examined by the Shapiro-Wilk’s test, normality plots and skewness/kurtosis statistics within each stage of Vieth. The age was summarized by minimum (min), maximum (max), mean ± SD, 95% confidence interval (CI) of mean, and median.

The age of males and females within each stage were compared by the Student’s t test or Mann-Whitney U test. The boxplots were drawn to show the age distribution. The inter- and intraobserver agreement for stage evaluation were assessed by the Cohen’s kappa and linear weighted kappa (κ, κ_w_) statistics since the stage is ordinal. The overall agreement rate and its 95% Wilson Score CI were also provided. A *p*-value < 0.05 was considered as statistically significant.

The cut-off values proposed by Landis and Koch [36] were used to interpret the κ and κ_w_ values as:

- κ < 0.20: *poor agreement*.
- κ = 0.21–0.40: *fair agreement*.
- κ = 0.41–0.60: *moderate agreement*.
- κ = 0.61–0.80: *good agreement*.
- κ = 0.81–1.00: *very good agreement*.

All statistical analysis and calculation were performed via IBM SPSS Statistics 25.0 (IBM Corp. Released 2017. IBM SPSS Statistics for Windows, Version 25.0. Armonk, NY: IBM Corp.).

## Results

**Fig. 3:**
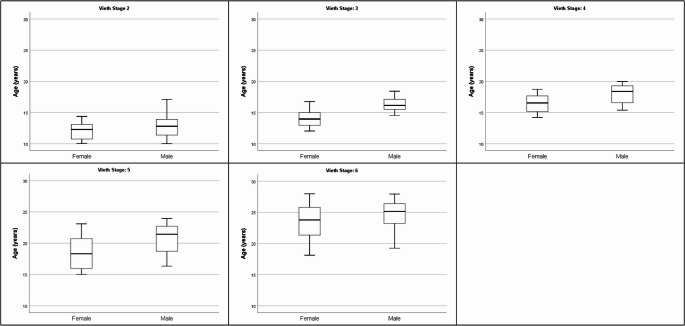
Age distribution of the females and males within each Vieth stage

The descriptive statistics for age in years (min-max, mean ± SD, 95% CI of mean, and median) are presented for females and males within each stage of Vieth in Table 2. The minimum ages at stages 2-3-4-5 were 10.06, 12.05, 14.22, 15.01, and 18.13 in females and 10.01, 14.54, 15.39, 16.34, and 19.26 in males, respectively. No statistically significant difference in age was observed between females and males at stage 2 (p-value = 0.166). The age of males was higher compared to the females in all other stages (p-values < 0.05, Fig. 3).

**Table 2:** Descriptive statistics of age based on sex and stage of Vieth

Stage	Sex	*n*	Min-Max	Mean ± SD	95% CI of Mean	Median	*p*-value
2	Female	14	10.06–14.40	12.09 ± 1.37	11.03–12.88	12.29	0.166^*^
	Male	19	10.01–17.13	13.06 ± 2.26	11.97–14.14	12.81	
3	Female	16	12.05–16.76	14.13 ± 1.34	13.41–14.84	13.97	**< 0.001** ^*^
	Male	18	14.54–18.44	16.39 ± 1.11	15.83–16.94	16.15	
4	Female	22	14.22–18.75	16.59 ± 1.43	15.96–17.23	16.54	**0.003**
	Male	26	15.39–19.98	18.05 ± 1.49	17.45–18.65	18.39	
5	Female	66	15.01–23.09	18.64 ± 2.53	18.02–19.26	18.31	**< 0.001**
	Male	69	16.34–23.95	20.75 ± 2.32	20.19–21.30	21.43	
6	Female	134	18.13–27.98	23.62 ± 2.66	23.17–24.08	23.78	**0.001**
	Male	132	19.26–27.95	24.71 ± 2.12	24.35–25.08	25.16	

*Min* Minimum, *Max* Maximum, *SD* Standard Deviation, *CI* Confidence interval

^*^Student’s t test was performed. Mann-Whitney U test was used for other stages

Bold values indicate statistically significant results (*p* < 0.05)

Intraobserver agreement was 0.935 (κw; agreement rate = 92.4%, 95% CI: 89.7–94.5) while interobserver agreement was 0.894 (κw; agreement rate = 88.4%, 95% CI: 85.2–91.0); all p-values were < 0.001. These statistics were substantially high, indicating very good inter- and intraobserver agreement for the evaluation of Vieth stages (Table 3).

**Table 3:** Intra- and interobserver agreements for the evaluation of the Vieth stage

	Intraobserver (*n* = 516)	Interobserver (*n* = 516)
κ ± se	0.886 ± 0.018	0.823 ± 0.022
κ_w_ ± se	0.935 ± 0.010	0.894 ± 0.014
Agreement rate (%) (95% CI)	92.4 (89.7–94.5)	88.4 (85.2–91.0)
*p*-value	**< 0.001**	**< 0.001**

*se* Standard error, *κ*_*w*_ Weighted kappa, *CI* Confidence interval

Bold values indicate statistically significant results (*p* < 0.05)

The results concerning the legally relevant age threshold of 18 years, which is critical for determining legal majority and related responsibilities, are presented in Table 4. In addition, Table 5 provides a comparison between our findings and those reported in previous studies that applied the Vieth method to the distal radial epiphysis [33–35].

**Table 4:** Distribution of male and female subjects according to stages and the 18-year legal age threshold

	< 18 (years)		≥ 18 (years)	
	Male	Female	Male	Female
Stage 2	19	14	0	0
Stage 3	16	16	2	0
Stage 4	12	19	14	3
Stage 5	14	29	69	37
Stage 6	0	0	132	134

**Table 5:** Comparison of studies performed with wrist MRI

Study	Study population and MRI protocol	Stage	Min-max ages	
			female	male
Ottow et al. (2022, Germany)	695 cases (12–24 years) 3.0 T MRI, cor T1-TSE, T2-TSE SPIR	2 3 4	12.11–13.75 12.3–17.82 14.44–23.98	12.05–17.07 13.69–19.15 15.35–20.94
		5 6	15.94–25 18.86–24.74	16.59–24.96 19.19–24.98
Ekizoglu et al. (2025, Turkey)	620 cases (10–30 years) 1.5 T MRI, cor T1-TSE, FS PD-TSE	2 3 4 5	10.08–15.58 12.33–16.58 14.25–19.92 16.33–22.75	9.92–15.67 15–18.5 15–20 17–22.75
		6	18.42–29.58	20–29.58
Ottow et al. (2026, Germany)	650 cases (12–24 years) 0.31 T MRI, cor PD-SPED	2 3 4	12.08–14.67 12.17–17.17 12.75–24.17	12–16.58 12–19.5 14.5–24.08
		5 6	15.58–24.67 17.25–24.92	16.67–24.92 18.42–24.83
This study (Turkey)	516 cases (10–28 years) 1.5 T MRI, cor T1-TSE, FS PD-TSE	2 3 4 5 6	10.06–14.4 12.05–16.76 14.22–18.75 15.01–23.09 18.13–27.98	10.01–17.13 14.54–18.44 15.39–19.98 16.34–23.95 19.26–27.95

## Discussion

In this retrospective study, distal radial epiphyseal fusion was evaluated on wrist MRI using the Vieth staging method in 516 individuals aged 10–28 years. The stages were assessed to determine the minimum and maximum age limits for each developmental stage in both sexes, and minimum age thresholds were established accordingly. High inter- and intraobserver agreement was observed, indicating the potential usefulness of this method for forensic age estimation, particularly at the legally relevant 15- and 18-year thresholds.

In our study, males were significantly older than females in Vieth stages 3–6, indicating that the same stages of distal radial epiphyseal fusion were reached at later ages in males. This finding reflects the well-established sex-related difference in skeletal maturation, whereby females generally experience earlier pubertal onset and faster epiphyseal maturation compared with males. Consequently, ossification and epiphyseal fusion tend to occur at younger ages in females, while the same developmental stages are reached later in males.

In the current literature, the application of the Vieth staging method to the distal radial epiphysis has been investigated in only a limited number of studies by Ottow et al. and Ekizoglu et al. [33–35]. In our study, intra- and interobserver agreement values (κw = 0.935 and κw = 0.894) indicate excellent reproducibility of the method using a 1.5 T MRI scanner with T1-TSE and FS PD-TSE sequences. Comparable levels of agreement have been reported in previous studies despite differences in magnetic field strength and sequence protocols. Ottow et al. [33], using 3.0 T MRI with T1-TSE and T2-TSE SPIR sequences, reported slightly lower agreement values (κ = 0.885 and κ = 0.872), while Ekizoglu et al. [34], who applied the same sequence protocol using 1.5 T MRI scanner as in our study, demonstrated even higher agreement (κ = 0.974 and κ = 0.961). Furthermore, in a low-field MRI study, Ottow et al. [35] reported κ values of 0.885 and 0.848 using 0.31 T MRI with Dixon-based PD sequences. Despite differences in scanner field strength and imaging protocols—including T2-TSE SPIR, FS PD-TSE, and Dixon-based PD sequences—observer agreement remained consistently high across studies. Nevertheless, given these methodological variations, such comparisons should be interpreted with caution when considering the broader applicability of the method in forensic age estimation. Although the original study employed T2-TSE SPIR sequences, which may offer advantages due to fat suppression and improved contrast resolution, we did not observe any clear limitations associated with the use of FS PD-TSE sequences in our retrospective dataset. Consistent with this observation, Gurses et al. reported that these two sequences can be used interchangeably for the evaluation of cartilage structures [26].

When comparing our findings with those of the other three studies based on the minimum age concept, our results were generally more consistent with those reported in the earlier study by Ottow et al. [33]. Due to variations in the minimum age of the study populations, evaluating the results for stage 2 presented certain challenges; for instance, the lower age limit in the cohorts studied by Ottow et al. [33, 35] was 10 years. In contrast, our findings indicated that the minimum ages for stages 3 to 5 in females were generally slightly lower than those in previous reports [33–35]. According to Ekizoglu et al., such disparities in minimum ages might arise from variations in sample size, population characteristics, and MRI-related methodological factors [34].

In a recent low-field MRI study, Ottow et al. [35] introduced a dedicated classification for assessing distal radial epiphyseal fusion using a 0.31 T MRI system with Dixon-based PD sequences. Although the stages showed a clear age-related progression, the minimum age for stage 6 in females was reported as 17.25 years, which lies below the legally relevant threshold of 18 years. According to the commonly applied minimum-age principle in forensic age estimation, a developmental stage with a minimum age below the legal threshold cannot be used as reliable evidence that an individual has already reached that age. Therefore, the authors concluded that the ultimate stage of their classification cannot reliably support the determination of legal majority in females. They also noted that the observed minimum ages may have been influenced by selection bias related to the predefined age range and the limited sample size, emphasizing the need for further validation in larger cohorts. In this context, comparisons with studies using different MRI field strengths and staging approaches remain important to clarify the applicability of distal radial MRI for forensic age estimation.

Some of the limitations of our study pertain to the lack of information regarding the socioeconomic status and ethnic background of the cases. It is a known fact that low socioeconomic status can delay bone development [37]. Regarding ethnic background, there are differing views in the literature. Some studies argue that ethnic background influences bone maturation [38], while others report that it does not, suggesting that socioeconomic conditions primarily affect bone development [39]. Based on this, we hypothesize that socioeconomic status plays a more significant role in bone maturation, and ethnic background does not have a distinct effect. Another limitation is the imbalanced age distribution between the sexes due to the retrospective design of the study. Additionally, our evaluations were performed using a 1.5 T MRI scanner. Although previous studies have reported comparable findings using different magnetic field strengths, including 3.0 T systems, potential differences in image quality and tissue contrast between scanners with different field strengths may influence the visualization of epiphyseal structures and should therefore be considered when interpreting and generalizing the results.

## Conclusion

The Vieth staging method allows reliable assessment of distal radial epiphyseal maturation on MRI with very good inter- and intraobserver agreement, supporting its use as a radiation-free tool in forensic age estimation. Using the minimum age concept, stage 5 was observed only above 15 years and stage 6 only above 18 years in both sexes, indicating applicability for the 15- and 18-year legal thresholds. However, further validation is required through future studies conducted in different populations with balanced age groups and consideration of sex and socioeconomic status, while methodological factors, including the MRI sequences used and the experience of observers, may also influence staging results.

## Abbreviations

κ: Unweighted kappa
κw: Linear weighted kappa
AGFAD: Arbeitsgemeinschaft für Forensische Altersdiagnostik
CI: Confidence interval
FOV: Field-of-view
FS: Fat-saturated
max: Maximum
min: Minimum
MRI: Magnetic resonance imaging
PD: Proton density
SD: Standard deviation
SE: Spin echo
SPED: Spin echo Dixon
SPIR: Signal pre-saturation with inversion recovery
T: Tesla
TE: Echo time
TR: Repetition time
TSE: Turbo spin echo
